# Hyaluronidase for microstomia in fibrosing skin disease: Systematic review and case series

**DOI:** 10.1016/j.jdin.2025.11.012

**Published:** 2025-11-28

**Authors:** Catherine Zhu, Ghassan Barnawi, Elen Grigorchuk, Ethan Bendayan, Katya Peri, François Lagacé, Houriah Y. Nukaly, Elena Netchiporouk

**Affiliations:** a Division of Dermatology, McGill University, Montreal, Quebec, Canada; b Faculty of Medicine, McGill University, Montreal, Quebec, Canada; c Faculty of Medicine, Université de Montréal, Montreal, Quebec, Canada; d Division of Experimental Medicine, McGill University, Montreal, Quebec, Canada

**Keywords:** hyaluronidase, microstomia, sclerosis, systematic review, trismus

*To the Editor*: Microstomia, characterized by reduced oral aperture, is a disabling feature of fibrosing skin diseases—such as systemic sclerosis (SSc), scleromyxedema, and mixed connective tissue disease—and may impair speech, mastication, or oral hygiene.^1^ Despite its functional and psychosocial impact, effective treatment options remain limited and inconsistently applied across clinical settings.^1^ Hyaluronidase, a glycosaminoglycan-degrading enzyme, may soften dense dermal matrix and improve perioral flexibility by enhancing local tissue permeability and reducing fibrosis.^2^ We conducted a retrospective case series and systematic review to evaluate its therapeutic efficacy and safety in microstomia associated with fibrosing dermatoses.

Eight adult patients with microstomia secondary to fibrosing connective tissue disorders treated with hyaluronidase at our tertiary dermatology center were retrospectively reviewed (McGill University Health Center Research Ethics Board #2021-7412). A systematic review (PROSPERO: CRD42024560040) was subsequently conducted in accordance with the PRISMA guidelines to identify studies evaluating hyaluronidase for microstomia related to fibrosing dermatoses. Screening was performed independently by 3 reviewers, and study quality was assessed using the Joanna Briggs Institute checklist (Supplementary Tables I and II, available via Mendeley at https://data.mendeley.com/datasets/xw8jhkrjr2/1).^3^^,^^4^ Statistical analysis was conducted using the Wilcoxon signed-rank test with continuity correction to compare vertical oral aperture and Mouth Handicap in Systemic Sclerosis scores before and after treatment.^5^ A *P*-value <.05 was considered statistically significant.

The median age of patients in our institutional cohort was 62.5 years (range, 41-69); 7 were women. Underlying etiologies of microstomia included limited SSc (*n* = 6), diffuse SSc (*n* = 1), mixed connective tissue disease (*n* = 1), and scleromyxedema (*n* = 1). All patients received intralesional hyaluronidase injections at a dose of 7.5 international units (IU) per injection site (total 150 IU per session), except for the patient with scleromyxedema, who received 15 IU per injection site (total 750 IU per session). Patients underwent a median of 2 treatment sessions (range, 1-4) spaced a median of 21 days apart (range, 21-65). All reported improvement, with a median increase in vertical mouth opening of 4 mm (range, 0-15) and a median Mouth Handicap in Systemic Sclerosis score improvement of 5 points (range, −4 to 10). Only the patient with scleromyxedema experienced recurrence of symptoms as his systemic disease progressed. No severe adverse events occurred. Injection discomfort was common with topical anesthetic alone, whereas patients receiving a combined nerve block and topical anesthetic reported minimal or no pain (Table I and Supplementary Table III, available via Mendeley at https://data.mendeley.com/datasets/xw8jhkrjr2/1).

**Table I:** Summary of our case series of 8 patients treated with hyaluronidase for microstomia

Patient	Anesthetic method	No of sessions	Total dose injected per session (IU)	Δ Vertical oral aperture∗	Δ MIHSS score†	Adverse events	Time to response (D)
(1) 61F, MCTD	Topical EMLA	2	150	+5 mm	−10	8/10 pain Mild bruising	21
(2) 47F, limited SSc	Topical EMLA	2	150	+8 mm	−7	None	21
(3) 57M, Scleromyxedema	Topical EMLA	4	750	+15 mm	+4 (worse)	6/10 pain	21
(4) 64F, limited SSc	Topical EMLA ± infraorbital/submental nerve block	3	150	+10 mm	−6	5/10 pain with EMLA None with nerve block	30
(5) 41F, limited SSc	Topical EMLA	3	150	No change	−1	6/10 pain	30
(6) 66F, limited SSc	Topical EMLA	1	150	+2 mm	Not available	8/10 pain Mild bruising	48
(7) 65F, diffuse SSc	Topical EMLA ± nerve block	1	150	+3 mm	−4	None	30
(8) 69F, limited SSc	Topical EMLA ± nerve block	1	150	+3 mm	−2	0/10 pain Mild bruising	18

*EMLA*, Eutectic mixture of lidocaine and prilocaine; *IU*, international units; *MCTD*, mixed connective tissue disease; *MIHSS*, Mouth Handicap in Systemic Sclerosis; *N*, number; *SSc*, systemic sclerosis; *Δ*, change in.

∗ ΔVertical oral aperture refers to the change in interincisal distance in millimeters (mm) before and after the last hyaluronidase injection session.

† ΔMIHSS refers to the change in MIHSS score before and after the last hyaluronidase injection session. The MIHSS is a scoring system used to characterize objectively the mouth handicap in SSc. It is based on a 12-item questionnaire that measures the degree of this handicap, with scores ranging from 0 to 48, where higher scores indicate greater mouth-related disability.

The systematic review identified 9 eligible studies: 5 case reports, 1 case series, and 3 retrospective cohorts, reporting on 28 patients (Supplementary Fig I, Table IV and V, available via Mendeley at https://data.mendeley.com/datasets/xw8jhkrjr2/1). When pooled with our cohort (total 36 patients), mean age was 55.2 years, 91.6% were female, and SSc accounted for 86.1% of cases (Supplementary Table VI, available via Mendeley at https://data.mendeley.com/datasets/xw8jhkrjr2/1). Hyaluronidase was delivered intralesionally in 35 patients (97.2%) and via iontophoresis in one case. Across studies, mean gain in vertical mouth opening was 7.9 ± 6.0 mm (*P* = .0057), and Mouth Handicap in Systemic Sclerosi improved by 8.8 ± 8.2 points (*P* = .0014), with an average time to response of 42.7 ± 25.4 days. No systemic or allergic reactions were reported (Table II).

**Table II:** Summary of hyaluronidase preparation and outcomes across the systematic review and institutional cohort

	Mean ± SD	*N* (%)
Hyaluronidase technique		
Intralesional injection (total)		35 (97.2)
Intradermal		25 (69.4)
Subcutaneous		10 (27.8)
Transmucosal		1∗(2.8)
Iontophoresis		1 (2.8)
Combination with ablative fractional laser (CO_2_)†		2 (5.6)
Combination with nonablative fractional laser (1540 nm)†		4 (11.1)
Use of anesthetics		
Topical anesthetic (EMLA)		19 (52.8)
Topical anesthetic and nerve block		7 (19.4)
None		8 (22.2)
Not recorded		2 (5.6)
Dose per injection point (IU) (*n* = 18)	8.8 ± 3.0	
Total dose per session (IU) (*n* = 33)	241.8 ± 164.3	
No of injection sessions (*n* = 35)	4.0 ± 4.3	
Interval between sessions (d) (*n* = 24)	43.3 ± 36.0	
Location (*n* = 30)		
Perioral		36 (100.0)
Cheeks		3 (8.3)
Glabella		1 (2.8)
Outcome measures		
Vertical oral aperture (interincisal distance, mm)		
Before (*n* = 16)	38.9 ± 9.9	
After (*n* = 16)	44.8 ± 8.4	
Δ (*n* = 32)‡	7.9 ± 6.0	
MHISS score (score out of 42)		
Before (*n* = 18)	29.9 ± 7.0	
After (*n* = 18)	21.1 ± 10.2	
Δ (*n* = 18)	8.8 ± 8.2	
Time to response (d) (*n* = 23)	42.7 ± 25.4	
Adverse events		
Transient pain during injection		21 (58.3)
Transient bruising		6 (16.7)

*CO*_*2*_, Carbon dioxide laser; *EMLA*, eutectic mixture of local anesthetics; *IU*, international units; *MHISS*, Mouth Handicap in Systemic Sclerosis; *N*, number; *SD*, standard deviation; *Δ*, indicates change from baseline.

∗ One patient received both intradermal and transmucosal injections.

† Some patients received multiple modalities or contributed to multiple categories.

‡ Thirty-two patients are reporting Δ but only 16 patients reported before and after interincisal distance.

These findings suggest that hyaluronidase is a promising, low-risk, and well-tolerated treatment for microstomia in fibrosing dermatoses. Although our case series is limited by small sample size, retrospective design, and protocol heterogeneity, the reproducible benefit across studies supports broader clinical adoption. Potential publication bias and variable study quality may also have influenced reported efficacy. Future prospective trials should define optimal injection techniques, treatment intervals, durability of response, and patient selection criteria to maximize outcomes.

## Conflicts of interest

Dr Netchiporouk has been a speaker or consultant, or has received investigator-initiated research funding from AbbVie, Arcutis, Bausch Health, Boehringer Ingelheim International, Bristol Myers Squibb, Galderma, Janssen, LEO Pharma, Medexus, Novartis Pharmaceuticals, Pfizer, Sanofi Genzyme, Sun Pharmaceuticals, Eli Lilly, and UCB. Dr Netchiporouk is the founder of Montreal Derm FilEZ website, which is a nonprofit educational resource. Other authors declare no conflicts. Drs Nukaly, Lagacé, Zhu, Barnawi, and Authors Peri, Bendayan, and Grigorchuk have no conflicts of interest to declare.
